# Successive site translocating inoculation potentiates DNA/recombinant vaccinia vaccination

**DOI:** 10.1038/srep18099

**Published:** 2015-12-15

**Authors:** Yanqin Ren, Na Wang, Weiguo Hu, Xiaoyan Zhang, Jianqing Xu, Yanmin Wan

**Affiliations:** 1 Shanghai Public Health Clinical Center, Fudan University, Shanghai, 201508, China; 2 Shanghai Cancer Center and Institutes of Biomedical Sciences, Department of Oncology, Shanghai Medical College, Fudan University, Shanghai, 200032, China; 3 Institutes of Biomedical Sciences, Shanghai Medical College of Fudan University, Shanghai, 200032, China

## Abstract

DNA vaccines have advantages over traditional vaccine modalities; however the relatively low immunogenicity restrains its translation into clinical use. Further optimizations are needed to get the immunogenicity of DNA vaccine closer to the level required for human use. Here we show that intramuscularly inoculating into a different limb each time significantly improves the immunogenicities of both DNA and recombinant vaccinia vaccines during multiple vaccinations, compared to repeated vaccination on the same limb. We term this strategy successive site translocating inoculation (**SSTI**). **SSTI** could work in synergy with genetic adjuvant and DNA prime-recombinant vaccinia boost regimen. By comparing *in vivo* antigen expression, we found that **SSTI** avoided the specific inhibition of *in vivo* antigen expression, which was observed in the limbs being repeatedly inoculated. Employing *in vivo* T cell depletion and passive IgG transfer, we delineated that the inhibition was not mediated by CD8^+^ T cells but by specific antibodies. Finally, by using C3^−/−^ mouse model and *in vivo* NK cells depletion, we identified that specific antibodies negatively regulated the *in vivo* antigen expression primarily in a complement depended way.

Preventing infectious diseases through vaccination is one of the major successes ever achieved in the history of public health. In spite of considerable progress that has been achieved, efficacious vaccine still remains elusive for pathogens that lack protective correlate or appropriate animal model, such as HIV, malaria and TB^1^,^2^. In efforts to develop effective vaccines against these “tough” pathogens, a couple of new vaccine modalities were introduced into the field, including DNA and viral vector-based vaccines, both of which are efficacious in animal models and have been licensed for veterinary applications^1^,^3^,^4^. In spite of being promising, neither DNA nor viral vector-based vaccine has ever been licensed for human use. The potential safety concerns and the pre-existing anti-vector immune responses constrain the translation from bench to bedside for viral vectors^5^,^6^. While, as being shown by dozens of clinical trials, the major limitation of DNA vaccine is its suboptimal immunogenicity^7^.

Many strategies have been attempted to improve it by targeting different aspects of DNA vaccination, which includes optimizing plasmid design, combining with traditional or genetic adjuvants, using next-generation delivering tools (such as electroporator, gene gun and bio-injector) and employing various prime–boost strategies. As being intensively investigated, both the genetic adjuvants (such as cytokines^8^,^9^ and cholera toxin^10^,^11^) and next-generation delivering tools^12^,^13^ showed high potency to improve the immunogenicities of DNA vaccines in animal experiments. Despite these encouraging pre-clinical evidences, the translation from animal models to human has proven to be very difficult. As being estimated, 100–1000-fold improvements of antibody titers that achieved by electroporation or cytokine adjuvants in mice may at best translate into a two- to three-fold improvement in humans^4^. And this is largely true according to the data of HVTN070/080 clinical trials, which showed that in spite of being capable of improving the overall responding rate, the combination of IL-12 plasmid and electroporation did not significantly enhance the magnitude of specific T cell responses^14^. Bio-injector(a needle free injection device) could induce higher responding rates in human^15^, however, a recent clinical study showed that although it could enhance the priming efficiencies of DNA vaccines, the differences in CD8^+^ T cell and antibody responses were less pronounced without rAd5 boosting^16^. Moreover, a recent randomized phase I clinical trial suggested that the bio-injector showed no superiority at improving the immunogenicity of rAd5 compared to needle injection^17^.

Confronted with these difficulties, we believe that it is still of high importance to either optimize the existed approaches or explore new ways to improve the immunogenicity of DNA vaccines. In this study, we report that successively changing the limb for intramuscular inoculation can significantly augment the immunogenicities of not only DNA vaccines but also recombinant vaccinia vaccines.

## Results

### Successively site-translocated inoculation (SSTI) significantly enhanced the immunogenicities of DNA vaccines, compared to anatomical site-fixed inoculation (SFI)

**Figure 1:**
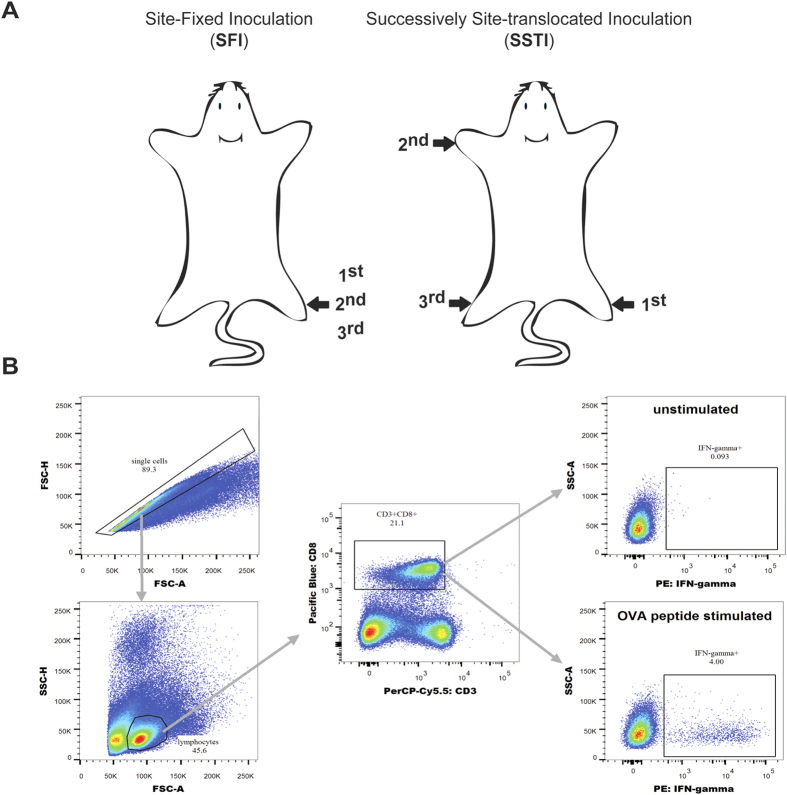
Schematic of vaccination strategy and Flow cytometry gating strategy. (**A**) (left) **SFI**: site-fixed inoculation, vaccines are injected into *tibialis anterior* on right-hind limb for 3 times. (right) **SSTI**: successively site-translocated inoculation, vaccines are injected into *tibialis anterior* on right-hind limbs for the 1^st^, left-fore limb for the 2^nd^, and left-hind limb for the 3^rd^ immunization. (**B**) Stained cells were first gated for singlets (FSC-H vs. FSC-A) and lymphocytes (SSC-A vs. FSC-A). Then the lymphocytes were gated based on the expression of CD3+ and CD8+ to identify CD8+ T cells. The expression of IFN-γ was calculated as: IFN-γ+CD8+% in cells stimulated with peptide minus IFN-γ+CD8+% in cells incubated with R10.

Two different inoculation modes were compared in this study: site-fixed inoculation (**SFI**) -mice were immunized by injection into *tibialis anterior* of the same limb, and successively site-translocated inoculation (**SSTI**) -mice were immunized by injection at different limb each time (Fig. 1A).

**Figure 2:**
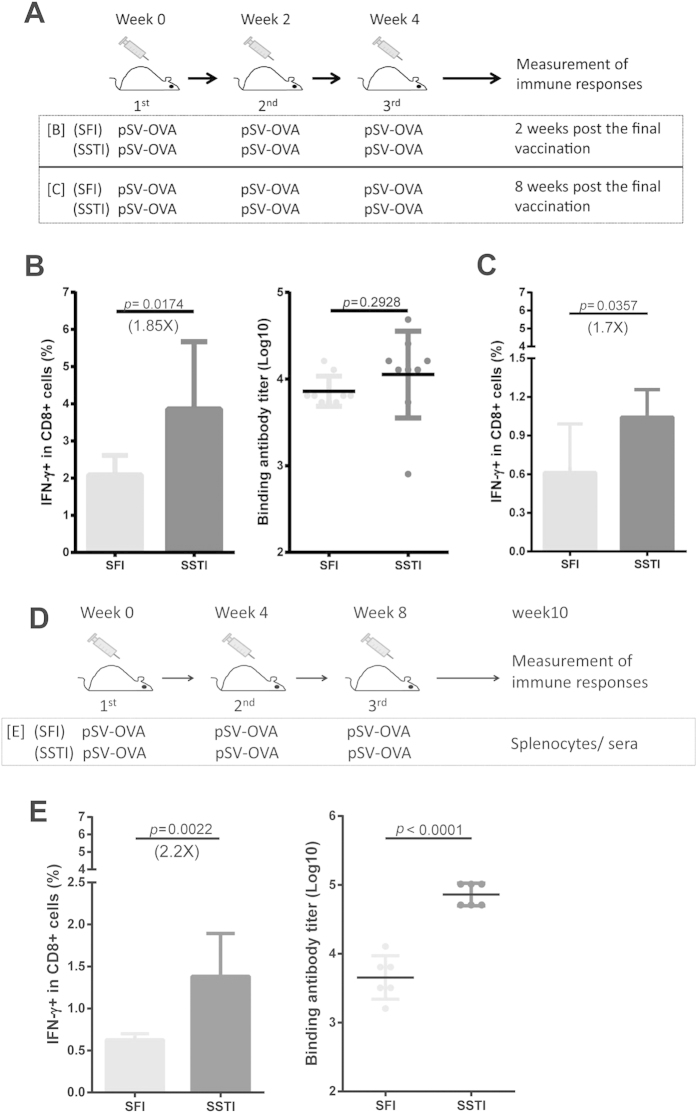
SSTI significantly enhanced immunogenicity of DNA vaccines both at effector and memory phases. (**A**) vaccination schedule. Antigen specific T cell responses (mean ± SD) were measured by ICS at both 2 weeks (**B**) and 8 weeks (**C**) post the last immunization (pSV-OVA). Specific binding antibodies (mean ± SD) (**B**) were detected by ELISA at 2 week post the last vaccination. Administrating the DNA vaccine at an interval of 4 weeks (**D**) also showed significantly higher T cell and antibody responses (**E**) in SSTI group. (B: n = 9; C: n = 6; E: n = 6)

C57BL/6 mice were immunized with pSV-OVA **SFI** or **SSTI** (Fig. 2A). 2 weeks after the last vaccination, mice were euthanized and splenocytes were harvested for measuring the specific CD8^+^ T cell responses by intracellular cytokine staining (ICS) (Fig. 2A). Our data showed that the average percentage of OVA_257–264_ specific IFN-γ secreting cells in CD8^+^ T cells elicited by **SSTI** was significantly higher than **SFI** (3.861 ± 1.809% vs 2.084 ± 0.5427%, *p* = 0.0174) (Fig. 2B). Although no significant difference was reached, the mean OVA-specific antibody titer induced by **SSTI** also tended to be higher than that of **SFI**. Next, to compare the T cell responses in memory phase, mice were euthanized for ICS assay at week 8 post the final vaccination (Fig. 2A). The results revealed that the OVA_257–264_ specific CD8^+^ T cell responses induced by **SSTI** (1.043 ± 0.214%) were also significantly higher than **SFI** (0.6133 ± 0.3769%, *p* = 0.0357) (Fig. 2C). Collectively, **SSTI** vaccinated group showed nearly 2 fold improvements in specific CD8^+^ T cell responses at both effector phase (2 weeks post the final immunization) and memory phase (8 weeks post the final immunization) , compared to **SFI** group.

Longer intervals between immunizations often seem to enhance quality and duration of immune response^18^. Herein, we immunized female mice with pSV-OVA **SFI** or **SSTI** in a 4-week interval (Fig. 2D), and 2 weeks after the last vaccination, immune responses were measured. The average percentage of OVA_257–264_ specific IFN-γ secreting cells in CD8^+^ T cells elicited by **SSTI** was significantly higher than **SFI** (1.382 ± 0.2086% vs 0.6268 ± 0.03014%, p = 0.0022) (Fig. 2E). And the mean OVA-specific antibody titer induced by **SSTI** was also significantly higher than that of **SFI** (p = 0.0001) (Fig. 2E).

### The improvement of immunogenicity was not due to the redistribution of specific CD8^+^ T cells and was not limited to OVA antigen

**Figure 3:**
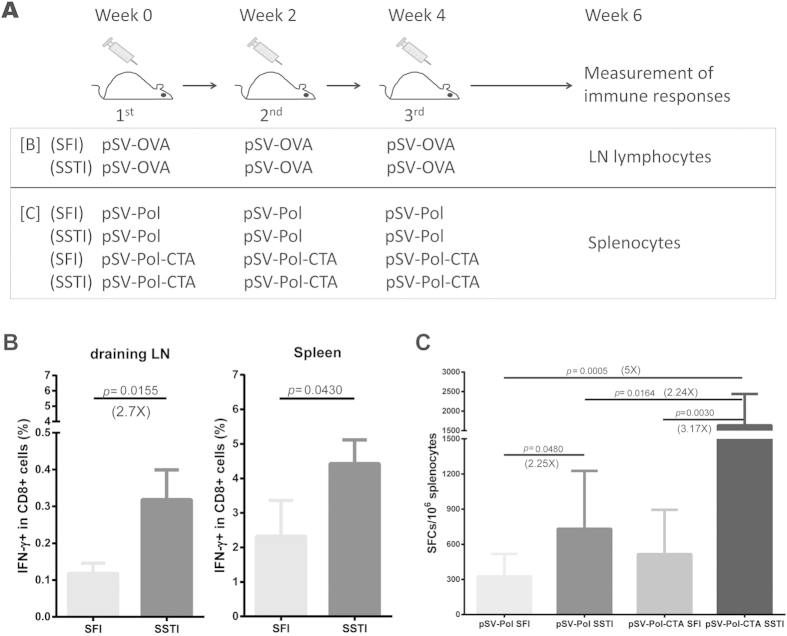
The improvement of immunogenicity was not due to the redistribution of specific CD8^+^ T cells and was not restricted to OVA antigen. (**A**) vaccination schedule. (**B**) 2 weeks post the 3^rd^ immunization(pSV-OVA), lymphocytes in draining lymph nodes and spleen were harvested and specific CD8^+^ T cells responses (mean ± SD) were measured by ICS (n = 3). (**C**) Specific T cell responses (mean ± SD) against HIV-1 Pol were detected by IFN-γ ELISPOT. SSTI also increased specific T cell responses induced by pSV-HIV-1 pol, and showed positive synergistic effect when combined with CTA adjuvant (n = 8).

To investigate whether the enhancement of cell-mediated immune response was due to the redistribution of specific CD8^+^ T cells, we collected the draining lymph nodes and spleens 2 weeks post the final vaccination. For each mouse, inguinal and iliac LNs were pooled together and the lymphocytes were isolated for ICS assay. Our data showed that **SSTI** induced significantly higher OVA_257–264_ specific CD8^+^ T cell responses in both draining LNs (*p* = 0.0155) and spleen (*p* = 0.0433) (Fig. 3A,B). The improvement seemed even more obvious in draining LNs.

We proceeded to explore whether our observations have a wide application. Immunization of mice with a DNA vaccine encoding full length Pol protein derived from HIV-1 AE2f also showed that total T cell responses elicited by **SSTI** were significantly higher than **SFI** (732 ± 495 SFCs/10^6^ splenocytes vs 325 ± 193 SFCs/10^6^ splenocytes, *p* = 0.048) (Fig. 3C). Moreover, our data showed that specific CD8^+^ T cell responses induced by **SSTI** could be further augmented by a genetic adjuvant (cholera toxin subunit A, CTA) (1638 ± 800 SFCs/10^6^ splenocytes), which was shown to be able to improve the immunogenicity of DNA vaccine in our previous work^11^ (Fig. 3C). This suggested that **SSTI** could work in synergy with other immunogenicity enhancing approaches.

### SSTI improved the magnitude of CD8^+^ T cell responses induced by both recombinant vaccinia alone and DNA-prime/recombinant vaccinia-boost, but not by protein vaccination

**Figure 4:**
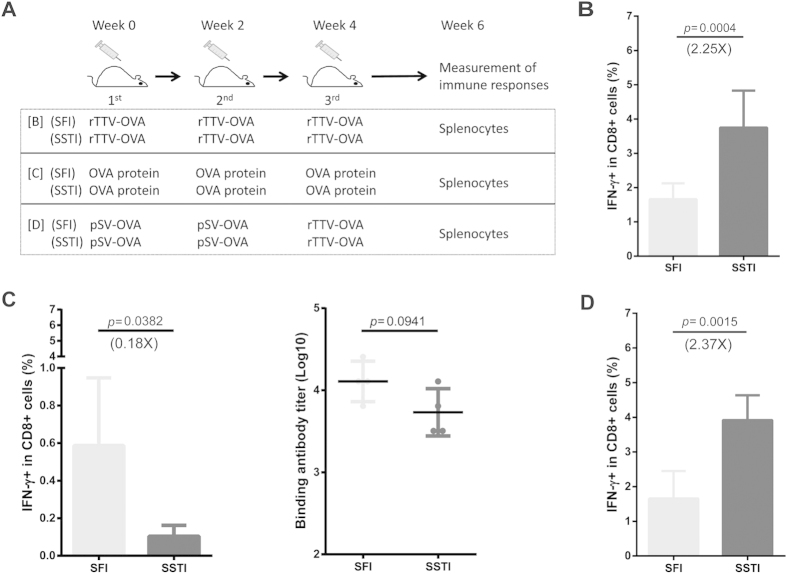
SSTI improved T cell responses induced by both recombinant vaccinia alone and DNA-prime/recombinant vaccinia-boost, but not by protein vaccination. Vaccination schedules for recombinant vaccinia, OVA protein and DNA prime-recombinant vaccinia boost were shown in (**A**). 2 weeks post the last immunization (rTTV-OVA), splenocytes were isolated and specific T cells responses were measured by ICS, binding antibodies were detected by ELISA. SSTI improved CD8^+^ T cell responses (mean ± SD) induced by recombinant vaccinia (**B**), however it decreased both the CD8^+^ T cell and binding antibody responses (mean ± SD) induced by OVA protein (**C**). SSTI significantly enhanced OVA-specific CD8^+^ T cell responses (mean ± SD) induced by DNA-prime/recombinant vaccinia-boost regimen (**D**). (B: n = 8; C: n = 4; D: n = 5)

To broaden our understanding of **SSTI** further, we tested whether it could also enhance the immunogenicities of vaccines in modalities other than DNA. Mice were immunized with three different regimens: recombinant vaccinia alone, DNA-prime/recombinant vaccinia-boost and OVA protein alone. For each regimen, two parallel groups were designed for the comparison of **SSTI** and **SFI** (Fig. 4A). 2 weeks after the 3^rd^ vaccination, the mice were euthanized and specific IFN-γ secreting CD8^+^ T cells were measured by ICS. Compared to **SFI**, **SSTI** augmented specific CD8^+^ T cell responses induced by recombinant vaccinia alone by 2.25-fold (3.751 ± 1.077% vs 1.664 ± 0.462%, *p* = 0.0004) (Fig. 4B) and augmented specific CD8^+^ T cell responses elicited by DNA-prime/recombinant vaccinia-boost by 2.37-fold (3.919 ± 0.7164% vs 1.656 ± 0.794%, *p* = 0015)(Fig. 4D). Very interestingly, when being immunized with purified OVA protein, the average specific CD8^+^ T cell responses induced by **SSTI** was significantly lower than **SFI** (0.1038 ± 0.05868% vs 0.5870 ± 0.3604%, *p* = 0.0382) (Fig. 4C). The same tendency was also observed in binding antibody titer, although no statistical significance was reached (Fig. 4C).

Taken together, the above data revealed that **SSTI** could augment the specific T cell responses induced by DNA and recombinant vaccinia vaccines, but not by protein vaccine.

### SFI significantly reduced the *in vivo* antigen expression compared to SSTI, in an antigen specific way

**Figure 5:**
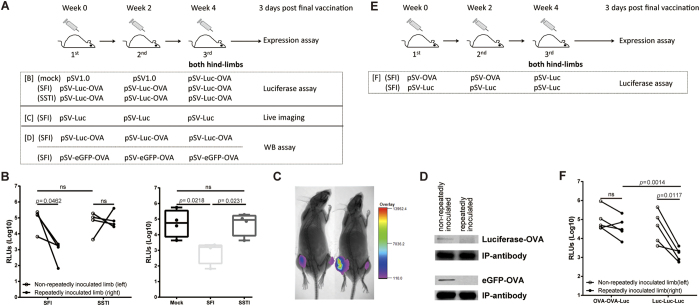
SFI significantly reduced the *in vivo* antigen expression in an antigen specific way. Vaccination schedules were shown in **(A,E)**. (**B**) The expression of Luciferase-OVA was measured by detecting luciferase activity, which showed the expression was significantly lower in the limb being repeatedly inoculated by SFI (n = 4). (**C**) Live imaging also showed the expression of Luciferase was much lower in the limb being repeatedly inoculated by SFI for 3 times. (**D**) The expression of Luciferase-OVA and eGFP-OVA were detected by western blotting after immunoprecipitation (IP-WB). The IP antibody (10 μg/sample) and the WB first antibody (diluted at 1:1000) was rabbit anti-OVA, and the WB second antibody was HRP-labeled goat anti rabbit (diluted at 1:5000). (**E,F**) The *in vivo* antigen expression was inhibited in an antigen specific way. Mice were immunized according to the schedule shown in (**E**). Luciferase expression was comparable between the repeatedly (right) and non-repeatedly (left) inoculated limbs in OVA-OVA-luc group, while, in luc-luc-luc group, the expression of Luciferase was much higher in the non-repeatedly inoculated limb(left) compared to repeatedly inoculated limb(right)(n = 5) (**F**).

The opposite influences on protein versus DNA/recombinant vaccinia vaccination implied that the *in vivo* antigen expression might be the key step that determined the difference between **SSTI** and **SFI**. To test this hypothesis, we inoculated mice with a luciferase expressing plasmid either by **SSTI** or **SFI** (Fig. 5A). Each mouse received two injections (one injection for each hind limb) for the 3^rd^ immunization. Three days after the 3^rd^ inoculation, *tibialis anterior* muscles of both hind limbs were isolated and homogenized for luciferase activity assay. Our data showed that the average luciferase activity in the repeatedly inoculated limbs of **SFI** group was significantly lower than that in the non-repeatedly inoculated limbs (paired *t-*test, *p* = 0.0462) (Fig. 5B). And it was also significantly lower than the average luciferase activity in the repeatedly inoculated hind limbs of control group, which received empty plasmids for the first two shots (*p* = 0.0218) (Fig. 5B). The average luciferase activities were similar between two hind limbs of **SSTI** group, both of which were significantly higher than the repeatedly inoculated limbs of **SFI** group (Fig. 5B). As there was no significant difference between the non-repeatedly inoculated limbs of **SFI** group and **SSTI** group, we then used the non-repeatedly inoculated hind limb of **SFI** group as the normal expression control in the following *in vivo* expression assays. *In vivo* live imaging also showed that the signal intensities of the repeatedly inoculated (right) limbs were obviously weaker than those of the non-repeatedly inoculated (left) limbs in **SFI** group (Fig. 5C). The observation was repeated by replacing the luciferase DNA with plasmids encoding luciferase-OVA or eGFP-OVA fusion protein and measured the *in vivo* expression by IP-WB. We found the expression of luciferase-OVA and eGFP-OVA were obviously decreased as well in the repeatedly inoculated limbs (Fig. 5D).

To further explore whether the reduction of antigen expression in the repeatedly inoculated limbs of **SFI** group was due to the pre-existing antigen-specific immune responses, we inoculated mice with OVA plasmid for the 1^st^ and the 2^nd^ time, but with luciferase plasmid for the last inoculation (Fig. 5E). Our data showed that the pre-vaccination of OVA didn’t interfere with the expression of luciferase, which indicated that **SFI** inhibited DNA vaccine expression in an antigen specific way (Fig. 5F).

### Compensating antigen expression by escalation the DNA dosage during site-fixed inoculation improved specific T cell responses to the level similar with SSTI

**Figure 6:**
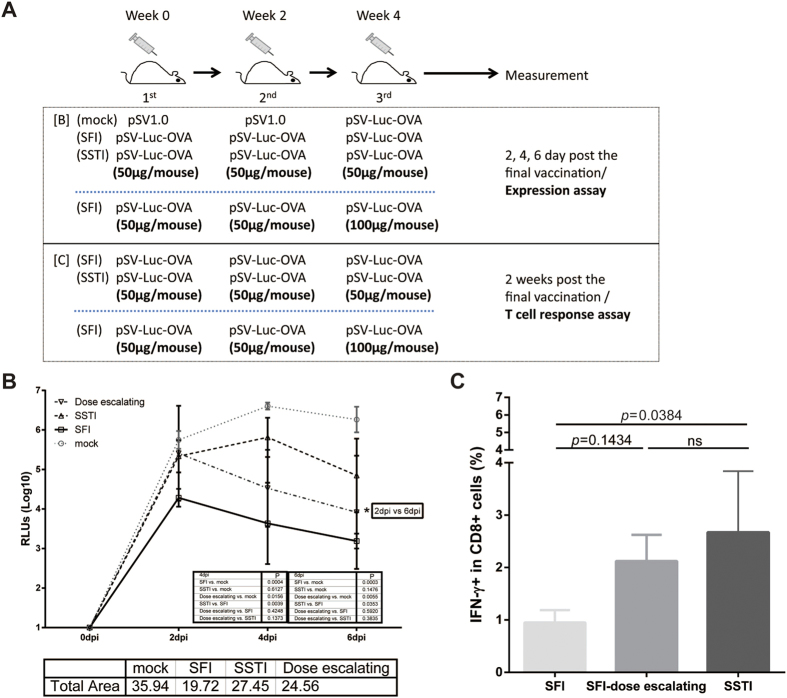
Escalation the DNA dosage compensated the antigen expression and improved specific T cell responses. Vaccination schedules were shown in (**A**). The dose of DNA vaccine was doubled for the 3^rd^ vaccination. (**B**) The kinetic of *in vivo* expression was measured by detecting luciferase activity in the inoculated limb at 2,4,6 days post the 3^rd^ vaccination. The order of AUC (area under curve) ranging from high to low was: mock > SSTI > dose-escalation > SFI (n = 3). (**C**) 2 weeks post the 3^rd^ immunization (pSV-OVA), splenocytes were isolated and OVA-specific IFN-γ secreting CD8^+^ T cells (mean ± SD) were measured by ICS (n = 5). Escalation the DNA dosage for the 3^rd^ vaccination narrowed the difference between SFI and SSTI group.

To clarify whether the reduction of *in vivo* antigen expression was the direct cause of decreased immunogenicity in **SFI** group, we designed a dose-escalation vaccination experiment (Fig. 6A), in which, each mouse received 50μg DNA for the first two shots and received 100 μg DNA for the 3^rd^ shot. *In vivo* antigen expression was monitored at 2, 4 and 6 days post the last vaccination. Ranging from high to low, the order of AUC (area under dynamic curve of *in vivo* expression) was: mock > **SSTI** > dose-escalation > **SFI** (Fig. 6B), suggesting that dose-escalation improved the *in vivo* antigen expression of the 3^rd^ shot in **SFI** group, but it was still lower than **SSTI** group. ICS assay revealed that dose-escalation improved the specific CD8^+^ T cell responses induced by **SFI**, although no statistical significance was reached (Fig. 6C).

### CD8^+^ T cells migrated to the inoculation site after the 3^rd^ immunization, but didn’t contribute to the reduction of antigen expression

**Figure 7:**
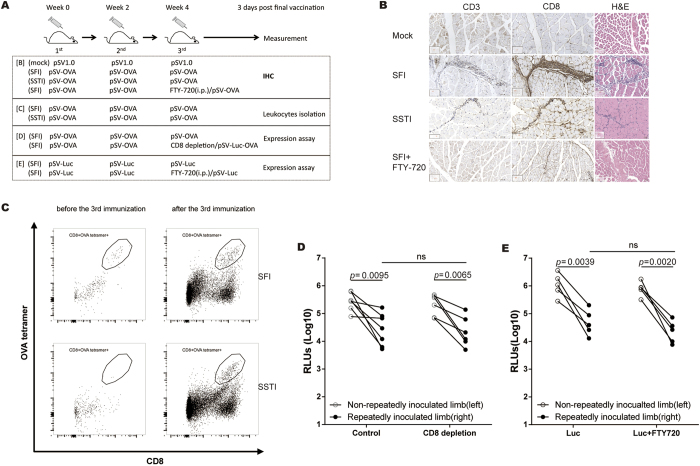
CD8^+^ T cells migrated to the inoculation sites, but didn’t contribute to the inhibition of antigen expression. 3 days post the last vaccination (**A**), *tibialis anterior* muscles were collected for hematoxylin-eosin (H&E) and histochemical staining. Much more CD8^+^ T cells migrated to the muscles in SFI group, and the migration could be blocked by administration of FTY720 (**B**). (**C)** leukocytes were isolated from *tibialis anterior* of the limbs that received the 3^rd^ immunization. Specific CD8^+^ T cells responses were detected by OVA tetramer^+^ staining. Depleting CD8^+^ T cells (**D**) or blocking the migration **(E)** before the 3^rd^ immunization did not counteract the reduction of antigen expression in the limbs being repeatedly inoculated. (D: n = 6/7; E: n = 5)

Three days after the 3^rd^ immunization, the *tibialis anterior* muscles of inoculation site were harvested for immunohistochemistry assay (Fig. 7A). Compared to **SSTI**, **SFI** attracted more infiltrated CD8^+^ T cell (Fig. 7B). And the infiltration could be largely blocked by administering FTY720 (Fig. 7B), indicating that the infiltrated cells were mainly migrated from the draining LNs. Further, we isolated the lymphocytes from the muscles of injection site 1 day before and 3 days after the 3^rd^ inoculation. Flow cytometry analysis showed that the OVA (SIINFEKL) tetramer^+^ CD8^+^ T cells were rare before the 3^rd^ vaccination, and their number increased sharply after the 3^rd^ vaccination. But, no significant difference was observed between **SFI** and **SSTI** (Fig. 7C).

To verify whether the infiltrated CD8^+^ T cells contributed to the reduction of *in vivo* antigen expression, we depleted mouse CD8^+^ T cells by giving depletion antibody intravenously one day before the 3^rd^ vaccination. Our data showed that the expression of luciferase in the leg that received 3 shots was still significantly lower than its expression in the other leg that received only one shot (*p* = 0.0065) (Fig. 7D). In a parallel experiment, mice were treated with FTY720, before the 3^rd^ vaccination to block the migration of CD8^+^ T cells. Consistently, we found the *in vivo* expression of luciferase wasn’t improved (Fig. 7E). These data implied that the reduction of *in vivo* antigen expression was not caused by specific CD8^+^ T cells.

### Specific antibodies led to the suppression of *in vivo* antigen expression in a complement dependent way

**Figure 8:**
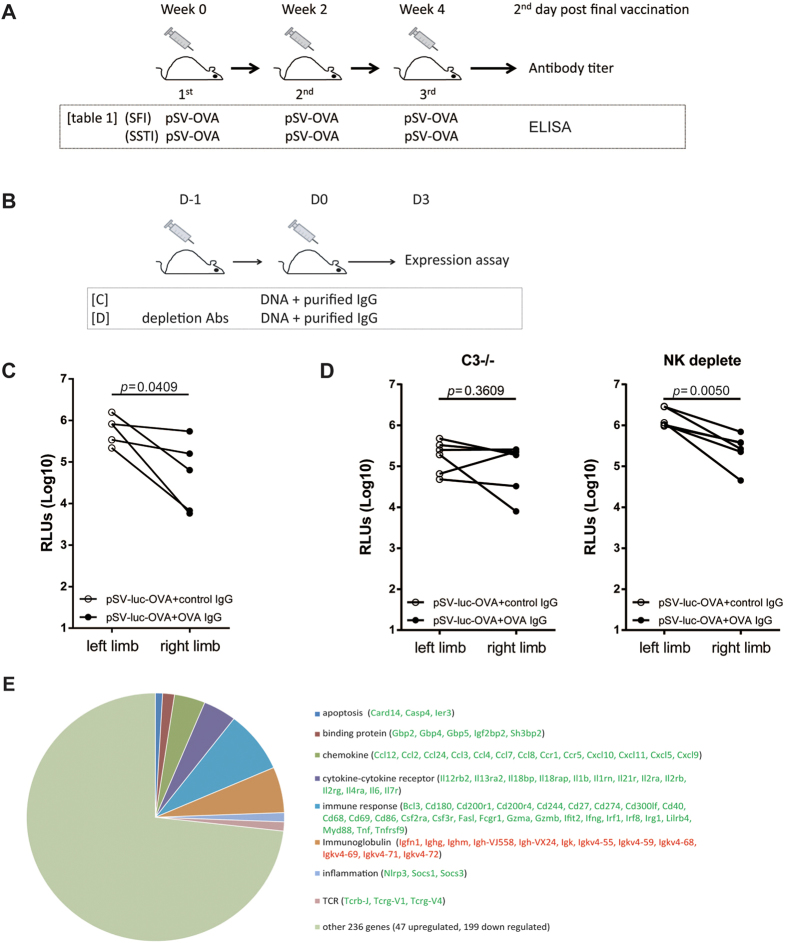
Specific antibody led to the suppression of *in vivo* antigen expression in a complement dependent way. Vaccination schedules were shown in (**A,B**). (**C**) pSV-Luc-OVA plasmids were mixed with purified anti-OVA IgGs or control IgGs and the expression Luc-OVA was detected by measuring luciferase activity. The presence of anti-OVA IgGs significantly decreased Luc-OVA expression (n = 5). (**D**) Complement component 3 knock out (C3^−/−^) mice and *in vivo* NK cell depletion were used to investigate the mechanism of antibody mediated expression reduction. C3^−/−^ mice showed comparable Luc-OVA expression between repeatedly (right) and non-repeatedly (left) inoculated limbs (n = 6), while NK cell depletion did not counteract antibody dependent suppression (n = 6). (**E**) Three days post the 3^rd^ vaccination, the injected muscles were isolated and total RNA was extracted for microarray analysis. Total 336 differentially transcribed genes were identified, including 67 upregulated (red ID) and 269 downregulated genes (green ID) (SFI vs SSTI, absolute fold change >2, *p* < 0.05, n = 3).

Ruling out the contribution of CD8^+^ T cell, then we were curious about whether specific antibody responses played a role in suppressing *in vivo* antigen expression, since the OVA specific antibody titers in the lysate *tibialis anterior* muscles of injection site were obviously higher in **SFI** group (Fig. 8A & Table 1). And this observation was supported by transcriptomic microarray analysis, which showed that the transcriptions of several Ig genes were significantly upregulated at the injection site of **SFI** group (Fig. 8E).

To test this hypothesis, we inoculated mice with the mixture of pSV-Luc-OVA DNA and purified serum IgG (Fig. 8B). For each mouse, 50μg pSV-Luc-OVA were mixed with 100μg total IgG freshly purified from either OVA vaccinated or empty plasmid vaccinated mice sera. Our data showed that total IgG purified from OVA vaccinated mice sera significantly suppressed the expression of luciferase-OVA (Fig. 8C), the average expression of luciferase decreased by >1 log10 (paired *t* test, *p* = 0.0409). The suppression could only be slightly counteracted by NK depletion, as our data showed that the average expression of luciferase still decreased by 0.76 log10 (paired *t* test, *p* = 0.005) in the limbs inoculated with the mixture of pSV-Luc-OVA and total IgG purified from OVA vaccinated mice sera (Fig. 8D). While, in C3^−/−^ mice, the suppression of *in vivo* luciferase expression could be almost completely counteracted (Fig. 8D), suggesting that the specific antibodies mainly functioned through a complement dependent way under this setting.

## Discussion

Over the past 20 years, DNA vaccines have proved effective in animal models^7^ and have been intensively evaluated in human trials^3^. Compared to conventional vaccines, DNA vaccines have a number of advantages, including ease of development and production, the ability to present antigen by both MHC class I and class II molecules^19^, and good safety record. However, an obvious disadvantage shown in human DNA vaccine trials has been their suboptimal immunogenicity when compared with traditional vaccines^7^. In spite of great efforts that have been made^4^, further optimizations are still needed to get the immunogenicity of DNA vaccine closer to the level required for human use.

In this study, we report for the first time that changing the inoculation site during multiple vaccinations (designated as successively site translocated inoculation, **SSTI**) can significantly enhance specific T cell responses elicited by DNA vaccines in both spleen and draining lymph nodes, compared to conventional anatomical site-fixed inoculation (**SFI**). While most previous efforts focused on the optimizations of plasmid design, codon preference, adjuvants, delivery tools or heterologous prime-boost regimens, our finding represents a new way to improve the immunogenicity of DNA and viral vector vaccines. Moreover, our data highlight that **SSTI** can work in synergy with genetic adjuvant (such as CTA) and DNA prime-recombinant vaccinia boost regimen. Several previous studies^18^,^20^ showed the immunization intervals significantly influenced the specific immune responses. Our data showed that **SSTI** also improved the immunogenicities of DNA vaccines that were administrated at an interval of 4 weeks.

Live attenuated and purified proteins are two of the major vaccine formalities under clinical use^21^,^22^, therefore, we also tested whether their immunogenicities could also be augmented by **SSTI**. A recombinant Tiantan vaccinia expressing OVA was used as an example of live attenuated vaccine and OVA protein was used as an example of purified protein vaccine. We found that **SSTI** significantly enhanced the immunogenicity of the recombinant Tiantan vaccinia. But to our surprise, the immunogenicity of OVA protein was weakened by **SSTI**, compared to **SFI**. These observations highlight the necessity of inoculating protein vaccine at the same site (limb), while imply that the DNA and viral vector vaccines should be injected at different limbs during multiple vaccinations.

Since the major difference between DNA/viral vector vaccines and protein vaccine is that the former need to express antigen *in vivo*, thus we postulated that giving DNA/viral vaccines by **SFI** may inhibit the *in vivo* antigen expression. And our data proved this hypothesis by showing that the expression of DNA vaccines after the 3^rd^ inoculation was significantly lower in the limbs that were repeatedly immunized. And we confirmed this is the leading cause of immunogenic difference between **SSTI** and **SFI**, because increasing the DNA dosage for the 3^rd^ inoculation could bring the immune responses induced by **SFI** to the level similar with **SSTI**. A reasonable explanation for this phenomenon is that the locally existed specific immune responses may inhibit the expression of “non-self” antigens encoded by DNA vaccines and our data supported this conjecture by showing that immunizing mice with pSV-OVA for the first two vaccinations did not interfere with the expression of luciferase for the third inoculation. It is quite similar with the mechanism that has been proposed for the immune surveillance of tumor^23^. But unexpectedly, our data showed that the inhibition is not mediated by T cells, but by specific antibodies. High titers of specific antibodies existed in the muscles which were repeatedly inoculated for 2 times or more, and these antibodies inhibited the later on expression of DNA vaccines in a complement dependent way. Of note, in addition to antigen specific antibody response, the anti-vector antibody response may also be an important factor that can restrict the replication of vaccinia and thus reduce its expression, which has been shown by several previous studies^24^,^25^,^26^,^27^.

To explain the difference of DNA vaccine expression between **SFI** and **SSTI**, we measured the antibody responses at the injection sites and our data showed that both the specific antibody titer and the transcriptions of Ig genes in **SSTI** group were much lower than **SFI** group (Table 1 & Fig. 8E), which we think is the main reason for the better expression of DNA vaccines in **SSTI** group. The presence of high Ig transcription level at the injection site of **SFI** group implied the existence of plasma B cells in the local environment, which might be due to the long lasting residual expression of DNA vaccine^28^. Besides, our microarray data also showed significantly different transcriptomic profiles at the injection sites of **SFI** group and **SSTI** group (Fig. 8E), which raised the possibility that the local microenvironment might contribute to the complement response. Another interesting finding of our study was that depleting NK cells only partially counteracted the suppression of *in vivo* antigen expression. Potential explanations for this phenomenon include: first, in addition to NK cell, myeloid cell (e.g. macrophage) can also mediate ADCC^29^,^30^; second, the suppression mediated by NK cells could be compensated to some extent by complements, as the complements are soluble factors and readily available in many tissues.

As most clinically used vaccines are injected into either side of two arms, we then tried to mimic this situation by optimizing **SSTI** into two limbs. However, we found that the expression of antigen was still obviously suppressed even when the DNA vaccine was inoculated into two different muscles of the same hind limb (data not shown), which was presumably due to the small figure size of mouse. Hence, we chose vaccination in three limbs as an alternative for this study. In spite of this limitation, our study suggests that changing the inoculation site (limb) successively (**SSTI**) during multiple vaccinations can improve the immunogenicity of both DNA and viral vector vaccines. But for protein vaccines and inactivated vaccines, inoculation at the same site is better, since they do not need to express antigens *in vivo*. We believe this strategy is of practical significance, especially when it can be properly used in combination with other immunogenicity enhancing approaches.

## Materials and Methods

### Ethics statement

Animal care and experiments were reviewed by the Institutional Animal Care and Use Committee (IACUC) of Shanghai Public Health Clinical Center and were performed in strict accordance with the approved protocol (Permit Number: 2013-E013). All experiments were performed at least two times with similar results. One representative result is shown.

### Reagents and vaccines

Mouse H-2K^b^ matched OVA peptide (SIINFEKL) was synthesized by Shanghai Science Peptide Biological Technology Co. ltd. HIV-1 AE2f Pol peptides (15-mers overlapping by 11 amino acids with its next peptide) were synthesized by GL Biochem (Shanghai) Ltd. The purity of all peptides was equal to or higher than 95%. H-2K^b^ OVA tetramer-SIINFEKL-APC was purchased from MBL (Cat# TS-5001-2C).

DNA vaccines expressing OVA, Luciferase, Luciferase-OVA, eGFP-OVA, HIV-1 AE2f Pol, or HIV-1 AE2f Pol-CTA were constructed previously by cloning the target genes into pSV-1.0 vector and all DNA vaccines used in this study were prepared by using the Endo-free Plasmid Giga Kit (Qiagen, Cat#12391) and reconstituted in sterile normal saline. Recombinant Tiantan vaccinia (rTTV) encoding OVA was also constructed in our previous work^10^. Purified OVA protein was purchased from Sigma (Sigma, Cat# A5378) and dissolved in sterile normal saline.

### Mice vaccination

6 to 8 week-old female C57BL/6 mice were housed under specific-pathogen free environment. All vaccines were injected intramuscularly (i.m.) and the detailed vaccination schedules and regimens were shown along with the results of each experiment. The dose of DNA vaccine was 50 μg/mouse except the 3^rd^ vaccination of the dosage escalation experiment in which 100 μg/mouse was used. The dose of recombinant vaccinia vaccine was 1 × 10^6^ pfu/mouse and the dose of protein vaccine was 20 μg/mouse.

### Intracellular cytokine staining

Freshly isolated splenocytes or lymphocytes were plated into round-bottom 96-well plates (2 × 10^6^ cells per well) and stimulated with OVA_(257–264)_(SIINFEKL) peptide at the final concentration of 5 μg/ml. 1 hour later, brefeldin A and monesin were added to each well at final concentration of 1 μg/ml and 1 μM. Another 7 hours later, the stimulation were stopped by washing the plates with R10 medium and 4 cell surface markers were stained on ice with fluorescein labeled antibodies, including PerCP-Cy5.5-labeled anti-mouse CD3 (Clone: 17A2, Biolegend Cat# 100218), Pacific Blue labeled anti-mouse CD8 (Clone: 53-6.7, Biolegend Cat# 100725), FITC-labeled anti-mouse CD44 (Clone: IM7, eBioscience Cat# 11-0441-81), and APC-eFluor780-labeled anti-mouse CD62L (Clone: MEL-14, eBioscience Cat# 47-0621-80). Next, the cells were fixed and permeablized, and intracellular IFN-γ was stained with PE-conjugated anti-mouse IFN-γ (Clone: XMG1.2, Biolegend Cat# 505808). Stained samples were measured using BD FACS Aria I. Data analysis was done by using FlowJo X software (Tree Star, Inc). Gating strategy was shown in Fig. 1B.

### IFN-γ ELISPOT assays

Enzyme-linked immunosorbent spot (ELISPOT) assays for IFN-**γ** release were performed by using mouse IFN-**γ** ELISPOT kits (BD Bioscience, Cat# 551083). The 96-well ELISPOT plates were coated with purified anti-mouse IFN-**γ** monoclonal antibody overnight at 4 °C. The plates were then blocked, and 2 × 10^5^ fresh splenocytes were added into each well and incubated with peptide for 20 h in a 37 °C 5% CO_2_ incubator. The final concentration for each peptide was 5 μg/ml. After incubation, detecting antibody and Avidin-HRP were added one after another. Subsequently, the plates were developed according to the manufacturer’s manual. Spots representing IFN-γ producing cells were enumerated by using an automated ELISPOT plate reader (ChampSpot III Elispot Reader, Saizhi, Beijing, China).

### ELISA

The serum antibodies against OVA were assessed using an enzyme-linked immunosorbent assay (ELISA) as previously described^11^. Briefly, 96-well EIA/RIA plates were coated overnight with 1 μg/ml of OVA protein at 4°C. Serially 2-fold diluted mouse sera were added to each well after being blocked with 200 μl 5% powdered milk. Binding Abs were detected by adding 1:2000 diluted goat anti-mouse IgG (HRP labeled) (Santa Cruz Biotechnology, Cat# sc-2005). After 1 hour incubation, 100 μl OPD substrate (Thermo Scientific, Cat# 34006) was added to each well. 15 minutes later, the reaction was stopped by adding 50 μl of 2N H_2_SO_4_. The endpoint antibody titers were defined as the last reciprocal serial serum dilution at which the absorbance at 492 nm was greater than 2× (negative mean + SD).

#### Antibody titer at the inoculation site.

was measured by ELISA. We isolated *tibialis anterior* muscle at 48 h post the last vaccination and homogenized with 600 μl Pierce IP Lysis Buffer (Thermo Scientific, cat# 87788). Supernatant was used for serial dilution (started from 1:10), and performed with the same method described above.

### Administration of FTY720

From the day of the 3^rd^ vaccination through **SFI**, mice were treated intraperitoneally every other day, with doses of 20 μg FTY720 (Cayman Chemical, Cat# 10006292) per mouse (1 mg/kg) in a final volume of 0.2 ml. The control mice were injected with 0.2 ml saline only^31^.

### *In vivo* luciferase expression analysis

Three days post the final pSV-Luciferase or pSV-Luciferase-OVA vaccination, C57BL/6 mice were euthanized, and the *tibialis anterior* muscle was excised and homogenized with 600 μl 1× luciferase cell lysis buffer (Promega, Cat# E1531). After 30 minutes incubation on ice, 50 μl supernatant of each sample was transferred to 96-well plate, following with 50 μl luciferase assay substrate (Promega, Cat#E1501). The relative luciferase unit (RLU) was detected with the GloMax^®^ 96 Microplate Luminometer (Promega Biotech Co., Ltd, Cat# E6521). RLU values were calculated according to normalized total protein concentration. All samples were tested in triplicate and the average RLUs were used in comparison of *in vivo* expression.

### *In vivo* imaging of luciferase expression

The mice were vaccinated with luciferase DNA through either **SFI** or **SSTI**. Luciferin for *in vivo* use(Promega, Cat# P1043) was injected into *tibialis anterior* muscle on the 3^rd^ day post last vaccination and ten minutes later, the mice were anaesthetized and the *in vivo* imaging was performed by using the *In-Vivo* Xtreme (Bruker Corporation, Cat#1815538).

### *In vivo* OVA expression assay

Three days after the final pSV-luciferase-OVA or pSV-eGFP-OVA vaccination, *tibialis anterior* muscles of the immunized mice were homogenized and lysed with Pierce IP Lysis Buffer. Rabbit anti-OVA polyclonal antibody was added to the supernatant, and the antibody/OVA complex was pulled down by Protein A+G agarose beads (Beyotime, Cat# P2012). The expression of luciferase-OVA or eGFP-OVA was determined by western blotting.

### Leukocytes isolation from *tibialis anterior* muscle

Three days post the final vaccination, C57BL/6 mice were euthanized and *tibialis anterior* muscles were excised and samples from mice in the same group were pooled together. Infiltrated lymphocytes were isolated according to a previously reported procedure with minor modification^32^. Briefly, the excised *tibialis anterior* muscle tissues were first washed with PBS and minced into small pieces. Then, the tissue pieces were digested in 0.2% collagenase IV (Life Technology, Cat# 17104-019) at 37 °C for 1 hour in a shaking incubator. Next, the tissue pieces were loaded and grinded in 70 μm nylon strainers. The passing through single cells were washed with R10 medium (RPMI-1640 supplemented with 10%FBS and 1%PS) and resuspended with 10 ml EZ-Sep^TM^ Mouse 1× lymphocytes isolation buffer (DAKEWE, Cat# DKW33-R0100). Isolation was completed following the manufacturer’s instruction. After a 500 xg centrifuge for 30 min, the leukocytes were harvested and washed with R10 twice, and then applied for fluorescent antibody and OVA tetramer staining.

### *In vivo* cell depletion

For CD8^+^ T cells depletion, 100 μg/mouse purified anti-mouse CD8 antibody (Biolegend, clone: 53-6.7, Cat# 100735) was injected via lateral tail vein 24 hours before the last vaccination. For NK depletion, 50 μg/mouse purified anti-Asialo-GM1 Antibody (Biolegend, clone: Poly21460, Cat# 146002) was injected through lateral tail vein 24 hours before vaccination.

### Serum IgG purification

Total IgG was purified from the sera of either pSV-OVA immunized mice or blank mice by using Protein G HP Spin Trap (GE Healthcare, Cat# 28-9031-34). And the OVA binding activities of the purified IgG were tested by ELISA.

### *In situ* detection of infiltrated CD8^+^ T cells

Three days after the final OVA DNA vaccination, the *tibialis anterior* muscles were excised and freshly fixed for immunohistochemistry assay. For each sample, three consecutive slides (thickness 4 μm) were stained with H&E, anti-CD3 and anti-CD8 mAb, respectively. The slides were scanned with Tissue FAXS Plus (Tissue Gnostics Gmbh, Austria).

### Statistical analysis

All statistical analyses were done by using GraphPad Prism 5.0 (GraphPad Software, Inc). Comparisons between two groups were analyzed by the method of *t* test. First check whether data follow normal distribution. If yes, *t* test is an appropriate measure of mean comparison. If not, then the Mann-Whitney test will be more appropriate for the same objective. Comparisons among three or more groups were done by One-way ANOVA. Significant difference was defined as *p* < 0.05.

## Additional Information

**How to cite this article**: Ren, Y. *et al.* Successive site translocating inoculation potentiates DNA/recombinant vaccinia vaccination. *Sci. Rep.* **5**, 18099; doi: 10.1038/srep18099 (2015).

## Figures and Tables

**Table 1:** OVA specific binding antibody titers in muscles.

	**SFI**		**SSTI**	
	**1 day before the 3**^**rd**^ **vaccination**	**3 days after the 3**^**rd**^ **vaccination**	**1 day before the 3**^**rd**^ **vaccination**	**3 days after the 3**^**rd**^ **vaccination**
**OVA-specific binding antibody titer**	80	40	<10	<10
	160	40	<10	<10
	20	160	<10	<10
